# Supplementary figures and images for: Development of spontaneous vegetation on reclaimed land in Singapore measured by NDVI
Source: PLoS One. 2021 Jan 28;16(1):e0245220. doi: 10.1371/journal.pone.0245220 (PMC7842902; doi:10.1371/journal.pone.0245220)

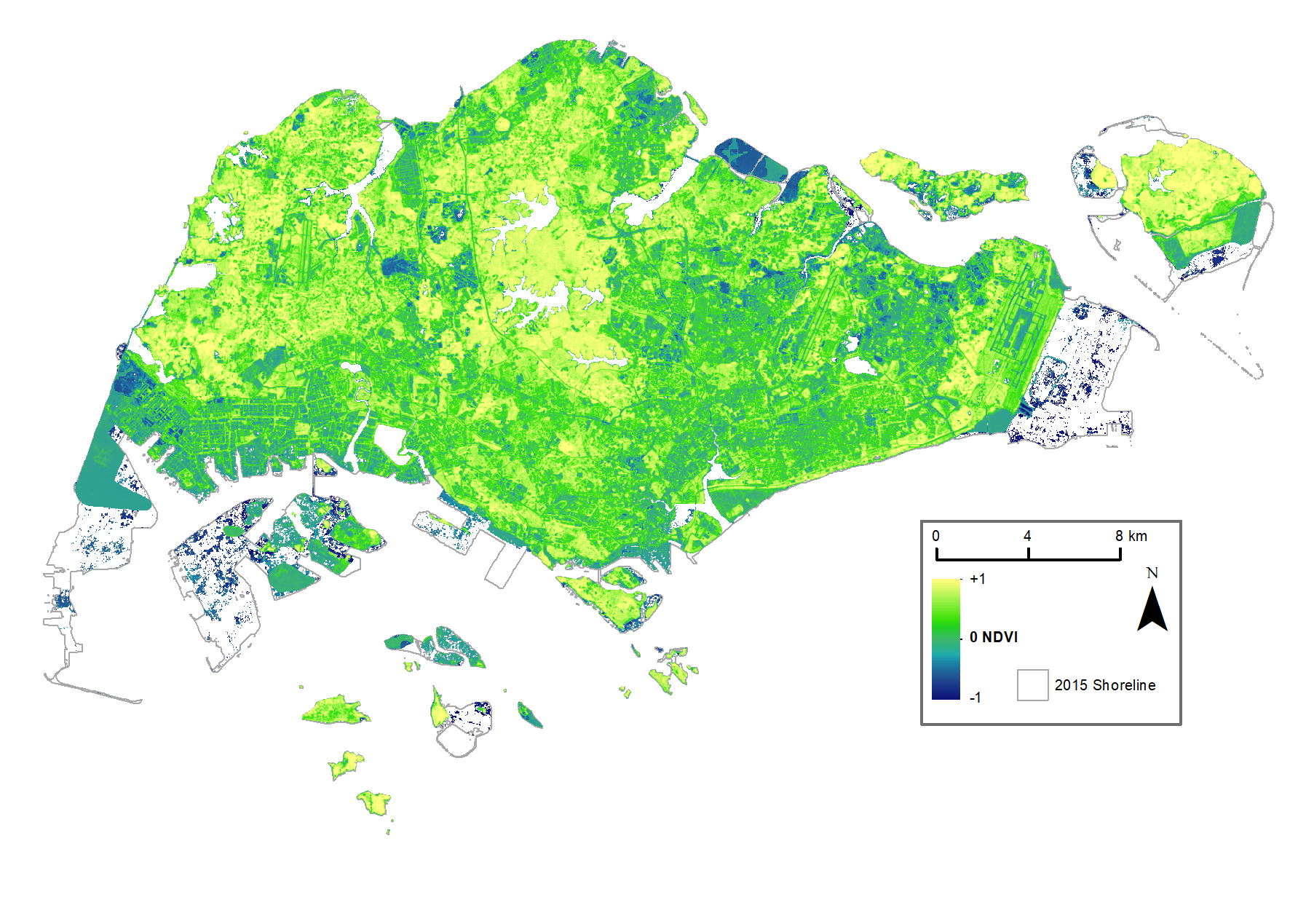

Supplement: S1 Fig — (TIF) [file pone.0245220.s001.tif]

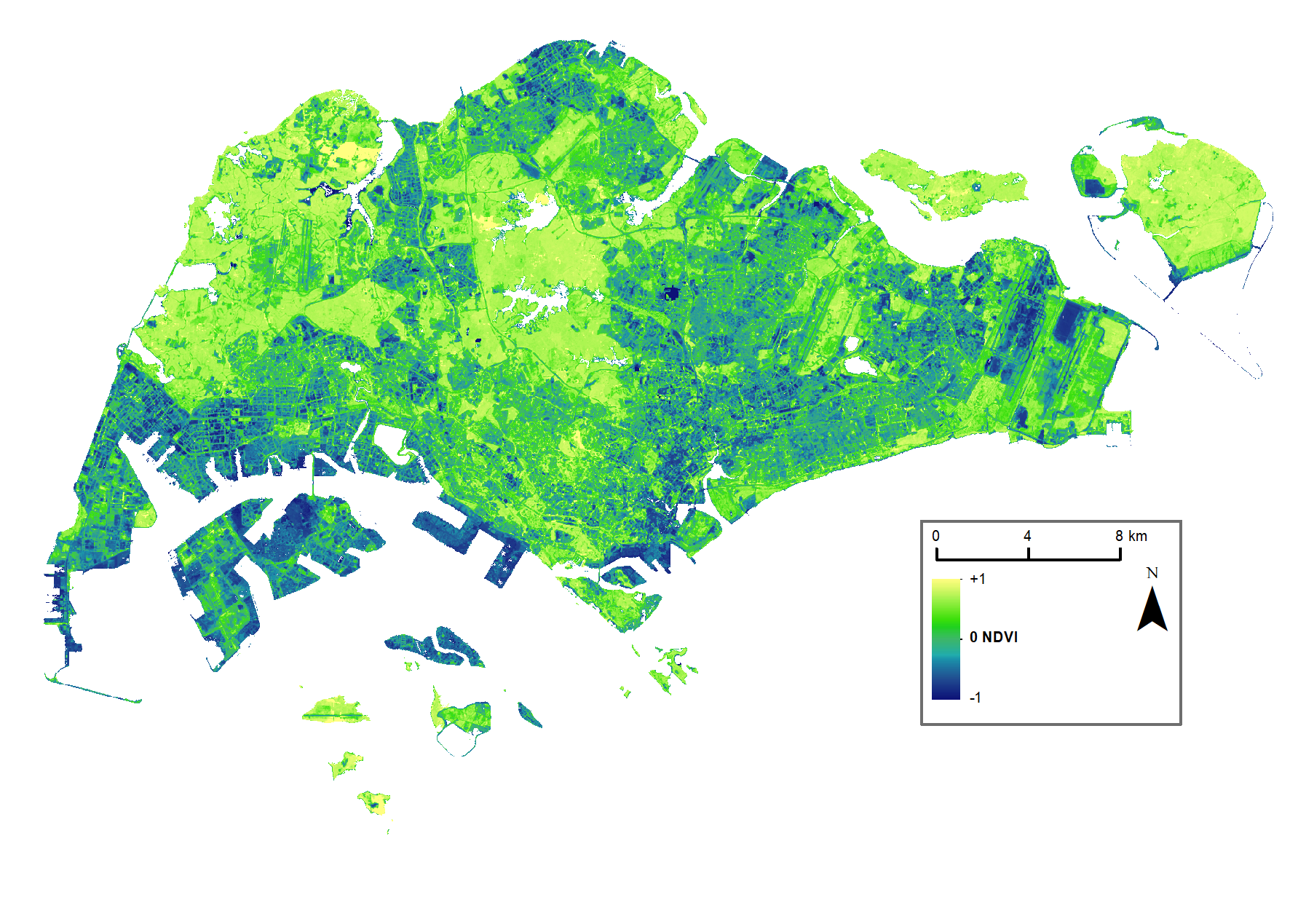

Supplement: S2 Fig — (TIF) [file pone.0245220.s002.tif]

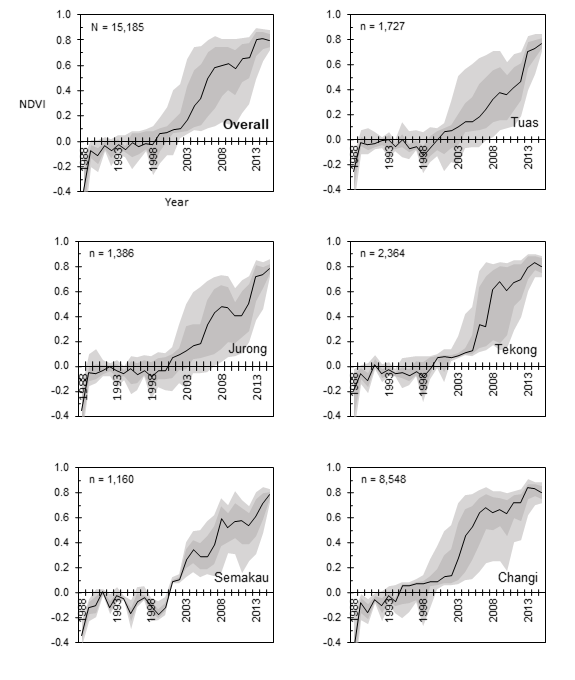

Supplement: S3 Fig — (TIF) [file pone.0245220.s003.tif]
