# Supplementary material for: Development of spontaneous vegetation on reclaimed land in Singapore measured by NDVI
Source: PLoS One. 2021 Jan 28;16(1):e0245220. doi: 10.1371/journal.pone.0245220 (PMC7842902; doi:10.1371/journal.pone.0245220)
Supplement: Attachment — Submitted filename: CoverLetter_Rev1.DOCX [file pone.0245220.s004.DOCX]

18^th^ November 2020

**Revised Manuscript PONE-D-20-26617: Development of spontaneous vegetation on reclaimed land in Singapore measured by NDVI**

Dear Professor Pickell,

On behalf of the co-author, I would like to thank you and the reviewers for your substantial efforts that has gone into reviewing our manuscript so far. Our detailed responses to these issues are listed in the *Response to Reviewer* section that follows this cover letter.

Thank you for the opportunity to respond to these reviews; the contribution of the reviewers has made this manuscript stronger, and we hope will make it of strong interest to the diverse audience of *PLOS One*, as well as a strong contribution to the broader land use dynamics literature.

Yours sincerely, on behalf of the co-author,

Leon Gaw

Research Associate

Natural Capital Singapore

ETH Zurich

**Response to Reviewers**

**Editor’s Comments**

There is no indication that the authors considered the confounding effect of temporal autocorrelation in their models. In the authors’ conceptual model of land reclamation, NDVI is temporally dependent. This leads me to believe that the Rsquared values of the models are overestimated and the p-values are difficult to interpret because the assumption of data independence has been violated. Please explain the temporal autocorrelation with an autocorrelogram and adjust your modelling techniques based on the autocorrelation structure of the data. Alternatively, the authors can try non-parametric models such as the Theil-Sen estimator and the Mann-Kendall rank sum test, which have both been extensively used and tested in the literature with NDVI time series.

*Response: We thank the reviewer for this suggestion. We have now added a first-order temporal correlation structure to the linear mixed-effects model, as suggested. This section has been updated in the methods (lines 181–190, Figure 5, and Table 3). The modelling results have changed slightly, most notably in adjusting the R-squared values slightly downwards, as would be expected.*

**Reviewer 1**

First of all, I think this is a very important paper and definitely should be published. The analysis and identification of natural growth areas will become increasingly important in the future and this paper further improves the knowledge needed to deal with these questions.

*Response: We thank the Reviewer for the positive comments and encouragement. We hope that our findings will highlight the importance of spontaneous vegetation growth on reclaimed lands, especially in expanding coastal cities.*

The arbitrary 0.7 threshold is ok and follows the values that normally are used for tropical areas. And the 15+ (16.8) years that have been found also follows the major scientific references like the ones studied by Robin Chazdon in her book "Second Growth: The Promise of Tropical Forest Regeneration in an Age of Deforestation" and by other important papers.

*Response: We have added Chadzon’s work as a reference and cited it in our manuscript line 177.*

Despite that, I have an issue with the results. The paper says that the NDVI in reclaimed areas has increased at a more rapid rate than the national median. But, the national median calculation also considers the reclaimed areas or not? Depending on the answer, I think it will be better to show the national median without those areas to have a real perception of the growths.

*Response: This suggestion is valid and we have included a trend line that shows the national median of NDVI that excludes newly reclaimed areas. Therefore, Figure 3 will have three NDVI trend lines of (1) the combined reclaimed and other terrestrial areas, (2) reclaimed areas only, (3) non-reclaimed terrestrial areas only. The slope for the total national and total national excluding reclaimed areas are similar.*

**Reviewer 2**

The manuscript does present a good overall view of the current status of Singapore's vegetation distribution

in specific areas. Using freely accessible datasets is also advantageous, keeps research open and reproducible. Methods used in the manuscript are straightforward and using the NDVI as a proxy to measure vegetation keeps matters simple.

*Response: We thank the Reviewer for the positive comments on our manuscript. We especially want to highlight the technological advances that have been made in remote sensing and how accessible they are now to researchers.*

Even though the paper is not using novel methods, it highlights important phenomena that affect the country, which is highly urbanised and built up, furthermore resulting in situations that are unique to Singapore and require targeted action (such as dealing with reclaimed land). The paper seems to use terms "Land use" and " land cover" interchangeably. The distinction needs to be made clear. In the Singaporean context, land use seems to make more sense, as most of the area is artificially built up and then it simply

begs the question what exactly is the land used for - for example, airport or housing. However, the background information section uses land cover only.

*Response: We agree with the Reviewer that the appropriate term in the urban context of Singapore is ‘land use’ and have standardised our manuscript to use only that term.*

The authors mention using Google Earth Engine, however, the script and code used was not attached. It makes sense to attach it in the supplementary material.

*Response: We will make our data and code available on a Figshare, an online repository of scientific work, once the manuscript is accepted for publication. This is in line with PLOS One’s open access policy.*

The authors also mention development of plant species on reclaimed land in the abstract and other parts in the paper. It would be meaningful to mention the exact type of vegetation growing on these reclaimed areas. The paper does highlight the ecological importance of the work, however, background information regarding the ecological state of the study area is somewhat lacking. Even though the aim of the paper is not to quantify vegetation, more background about the type of plant species and implications for ecology should be highlighted more.

*Response: We have added a discussion section to elaborate on the plant ecology and its state in reclaimed lands in Singapore (lines 287–307).*

Also English requires some revision, there is a lot of switching between active and passive voice throughout.

*Response: We have read through the manuscript again and have standardised our writing to a passive voice writing style.*
